# Coccidioidal Pneumonia, Phoenix, Arizona, USA, 2000–2004 

**DOI:** 10.3201/eid1503.081007

**Published:** 2009-03

**Authors:** Michelle M. Kim, Janis E. Blair, Elizabeth J. Carey, Qing Wu, Jerry D. Smilack

**Affiliations:** Mayo Clinic, Scottsdale, Arizona, USA; 1 Current affiliation: Kirksville College of Osteopathic Medicine, Kirksville, Missouri, USA.

**Keywords:** Coccidiodomycosis, Coccidioides, mycoses, pneumonia, bacterial, research

## Abstract

Community-acquired pneumonia (CAP) often results in severe illness and death. In large, geographically defined areas where *Coccidioides* spp. are endemic, coccidioidomycosis is a recognized cause of CAP, but its frequency has not been studied extensively. To determine the frequency of patients with coccidioidomycosis, we conducted a prospective evaluation of 59 patients with CAP in the Phoenix, Arizona, area. Of 35 for whom paired coccidioidal serologic testing was performed, 6 (17%) had evidence of acute coccidioidomycosis. Coccidioidal pneumonia was more likely than noncoccidioidal CAP to produce rash. The following were not found to be risk factors or reliable predictors of infection: demographic features, underlying medical conditions, duration of time spent in disease-endemic areas, occupational and recreational activities, initial laboratory studies, and chest radiography findings. Coccidioidomycosis is a common cause of CAP in our patient population. In the absence of distinguishing clinical features, coccidioidal pneumonia can be identified only with appropriate laboratory studies.

## Abstract 2

A prospective evaluation identified *Coccidioides* spp. as frequent causes of community-acquired pneumonia.

Coccidioidomycosis is caused by infection with *Coccidioides* spp., which consist of the nearly identical *Coccidioides immitis* and *C*. *posadasii* that grow in the soils of the desert southwestern United States and in limited areas of Central and South America (*1*). When soil is disrupted, airborne arthroconidia can be inhaled, causing infections in humans and animals. These infections can be asymptomatic or can produce illness of varying severity, from mild, self-limited respiratory infection to severe, life-threatening pneumonia (*2*). In a small percentage of patients, *Coccidioides* spp. may spread beyond the pulmonary tract, most frequently to the cutaneous, osteoarticular, or central nervous systems (*2*).

The incidence of coccidioidomycosis has increased dramatically from 2.5 cases/100,000 persons in 1996 to 8.4 cases/100,000 persons in 2006 in California (*3*) and from 21 cases/100,000 in 1997 to 91 cases/100,000 in 2006 in Arizona (*4*). Clinicians in disease-endemic areas are usually aware of coccidioidomycosis but often do not consider the diagnosis in patients who initially have respiratory symptoms (*5*). A possible explanation for this oversight may be uncertainty about the frequency of coccidioidomycosis as a cause of acute community-acquired pneumonia (CAP). Valdivia et al. (*5*) reported that 29% of patients with CAP in Tucson, Arizona, had coccidioidomycosis. To determine the frequency of coccidioidal pneumonia in a second sample of the population, we studied patients with CAP in the Phoenix, Arizona, metropolitan area.

## Methods

We evaluated patients with acute CAP who were admitted to our hospital or who sought care in the emergency department or ambulatory family practice and internal medicine outpatient clinics of our institution’s multispecialty referral practice and primary care practice in the Phoenix area. Patients were enrolled during February 2000–November 2004. This study was reviewed and approved by the Mayo Clinic Institutional Review Board.

Patients were eligible for enrollment if they had acute signs and symptoms of pneumonia (including but not limited to fever, cough, dyspnea, chills, and rash) and radiographic evidence of a pulmonary infiltrate identified by an independent radiologist not on the study team. Patients were excluded if they were <18 years of age, were already receiving oral or parenteral antifungal treatment, had radiographically documented pneumonia predating the current illness but within the past 3 months, were unable to return for subsequent serologic tests, or were otherwise unwilling to provide informed consent. Patients were also excluded if the evaluating physician had initially suspected coccidioidomycosis and had already ordered a serologic test for *Coccidioides* spp. as part of the initial clinical evaluation.

After goals and requirements of the study were explained to patients and informed consent was obtained, a pen-and-paper questionnaire was given to each patient for completion. The questionnaire collected information about patients’ residence in disease-endemic regions, occupations, daily activities, and previous medical conditions. Within 24–48 hours of enrollment, participants had blood samples collected for serologic tests; these tests were repeated 6–8 weeks later. After all patients completed enrollment, we analyzed the patients’ clinical signs and symptoms and results of laboratory studies (including serologic studies, complete blood cell counts, erythrocyte sedimentation rates, eosinophil counts, and culture results, if obtained), chest radiographs, medical treatment, and follow-up data.

Serologic tests for *Coccidioides* spp. were enzyme immunoassay (EIA), immunodiffusion (ID), and complement fixation (CF) tests. Qualitative detection of immunoglobulin (Ig) M and IgG to *Coccidioides* spp. by using EIA was performed by using a commercially available test kit (Meridian Bioscience, Inc., Cincinnati, OH, USA). ID and CF antibody tests were performed at the laboratory of Dr D. Pappagiannis (University of California Medical Center, Davis, CA, USA).

For this study, a case of coccidioidomycosis was defined by the presence of acute signs or symptoms of respiratory infection (e.g., cough, fever, pleuritic or chest pain, or dyspnea) in combination with a radiographically demonstrated pulmonary infiltrate and positive results from paired (initial and follow-up) coccidioidal serologic tests. We considered results of paired serologic tests to be positive if we observed 1) seroconversion (an initial negative serologic result followed by a positive serologic result); 2) an initial positive serologic result followed by an increase in the number of positive qualitative test results among serologic methods (e.g., initial EIA IgM positive, IgG negative, ID negative, CF negative followed by a positive result of >1 of the following: EIA IgG, ID, CF); or 3) an increase in serologic titer on the second CF test.

Differences in distributions of dichotomous variables were analyzed by using the χ^2^ test or the continuity-adjusted χ^2^ test when appropriate. The Fisher exact test was used for comparisons with small sample size. Differences between distributions of continuous variables were analyzed by using the independent *t* test. Data analysis was performed by using the statistical software program SAS version 9.1.3 (SAS Institute, Inc., Cary, NC, USA).

## Results

During February 2000–November 2004, 62 patients were enrolled in the study, 3 of whom were subsequently excluded from analysis because of incomplete questionnaires and lack of serologic results. Of the remaining 59 patients, 35 completed the requirement for paired coccidioidal serologic testing. For adequate statistical power, we wanted to enroll 175 patients. However, because of the slow accrual of patients, the study ended before we reached this target enrollment. Serologic results for the 59 study participants are summarized in Table 1.

**Table 1:** Serologic test results for *Coccidioides* spp. in 59 patients, Phoenix, Arizona, USA, 2000–2004*

No. patients	First serologic result					Second serologic result				
	EIA IgM	EIA IgG	ID	CF		EIA IgM	EIA IgG	ID	CF	CF titer
Negative serologic results: completed 2 tests										
11	–	–	–	–		–	–	–	–	
5	ND	ND	–	–		ND	ND	–	–	
3	ND	ND	–	–		–	–	–	–	
6	–	–	ND	ND		–	–	–	–	
1	–	–	–	–		ND	ND	–	–	
1	–	–	ND	ND		–	–	ND	ND	
1	–	–	ND	ND		ND	ND	–	–	
1	–	–	–	–		–	–	ND	ND	
Negative serologic results: completed 1 test										
10	ND	ND	–	–		ND	ND	ND	ND	
4	–	–	ND	ND		ND	ND	ND	ND	
6	–	–	–	–		ND	ND	ND	ND	
Positive serologic results: completed 2 tests										
2	–	–	–	–		+	–	+	–	
1	ND	ND	–	–		+	–	+	+	4
1	ND	ND	–	–		–	+	–	–	
1	ND	ND	–	–		–	–	+	–	
1	–	–	–	–		–	+	+	+	16
Positive serologic results: completed 1 test										
3	+	–	–	–		ND	ND	ND	ND	
1	ND	ND	ND	ND		–	–	–	+	2

*EIA, enzyme immunoassay; Ig, immunoglobulin; ID, immunodiffusion; CF, complement fixation; ND, not done; –, negative test result; +, positive test result.

All 35 patients who completed paired serologic testing were white, and 15 (43%) were men (Table 2). For 6 (17%) of the 35 patients (95% confidence interval [CI] 7%–34%), a diagnosis of coccidioidomycosis was based on coccidioidal antibody seroconversion. Patients with coccidioidal infection were more likely than those with noncoccidioidal CAP to have rash (p = 0.002). Among 6 patients with coccidioidomycosis, no association was found between coccidioidal infection and patient’s sex or race, symptoms other than rash, findings on chest radiographs, or laboratory data. Coccidioidal infection had no association with specific medical conditions, occupations, or recreational activities. Patients who had coccidioidomycosis had lived somewhat less time in the disease-endemic area (mean years of residency 14.6 years, range 2.5–26 years) than had patients whose pneumonia was caused by other factors (mean years of residency 25.2 years, range 0.5–69 years); this difference was not statistically significant.

**Table 2:** Characteristics of 35 CAP patients with or without coccidioidomycosis, Phoenix, Arizona, USA, 2000–2004*

Characteristic	No. patients		p value
	With coccidioidomycosis (n = 6†)	Without coccidioidomycosis (n = 29†)	
Age, y, mean (range)	59.2 (38.0–80.3)	67.8 (61.8–73.8)	0.25
Male sex	2 (33)	13 (45)	0.60
White race	6 (100)	27 (93)	0.50
Location where evaluated			
Hospital	4 (67)	20 (69)	0.99
Emergency department	1 (17)	3 (10)	0.55
Outpatient clinic	1 (17)	6 (21)	0.99
Signs and symptoms			
Cough	4 (67)	27 (93)	0.12
Sputum production	1 (17)	16 (55)	0.18
Dyspnea	6 (100)	18 (62)	0.21
Fever	5 (83)	21 (72)	0.99
Chills	3 (50)	11 (38)	0.98
Night sweats	2 (33)	7 (24)	0.99
Rash	3 (50)	0	0.002
Chest radiograph findings			
Unilateral infiltrates	5 (83)	17 (59)	0.50
Bilateral infiltrates	1 (17)	8 (28)	0.96
Laboratory results			
Leukocyte count, ×10^9^ cells/L, mean (range)	15.1‡ (9.1–21.2)	11.0§ (9.1–12.9)	0.07
Absolute eosinophil count, ×10^9^ cells/L, mean (range)	2.96¶ (0.18–13)	0.12# (0.0–0.2)	0.32
Empiric coccidioidal treatment prescribed after initial visit	1 (17)	1 (3)	0.76

*CAP, community-acquired pneumonia. Values are no. (%) unless otherwise indicated. †Unless otherwise indicated. ‡n = 6. §n = 25. ¶n = 5. #n = 22.

Of the original 59 patients enrolled, 24 were excluded because they lacked 1 of the 2 blood samples required for paired serologic testing. To ascertain whether bias was introduced by this exclusion, we compared demographics, symptoms, and laboratory and radiographic findings of the excluded patients with those of the 35 patients who remained in the study. The 24 excluded patients were more likely to have unilateral infiltrates (22 [92%] of 24 vs 22 [63%] of 35; p = 0.01). No other significant differences were identified.

## Discussion

Pulmonary coccidioidomycosis is a febrile respiratory illness with symptoms similar to or identical to those of nonmycotic CAP. Common features include fever, headache, cough, chest pain, dyspnea, and fatigue. This similarity in symptoms makes it difficult to recognize coccidioidal infection in the absence of diagnostic tests. Because most patients with primary coccidioidal pneumonia have spontaneous resolution of signs and symptoms, a patient with undiagnosed coccidioidomycosis who receives antibacterial therapy may appear to respond to treatment. However, a substantial portion of patients with coccidioidal pneumonia may have a protracted course and may benefit from specific antifungal treatment (*6*). Two of the authors (J.E.B. and J.D.S.) have observed that medical practitioners in the Phoenix metropolitan area often administer empiric antibacterial treatment to patients with CAP and test for coccidioidomycosis only when treatment fails (unpub. data). This combination of factors is likely to lead to underestimation and underappreciation of the likelihood of coccidioidomycosis as a cause of acute CAP.

 In the current study, we diagnosed coccidioidal infection in 6 (17%) of 35 (95% CI 7%–34%) patients in the Phoenix metropolitan area. Similarly, near Tucson, Arizona, coccidioidomycosis had been identified in 16 (29%) of 55 patients (95% CI 6%–44%) who sought treatment for CAP (*5*).The CIs associated with these incidence estimates overlap considerably. If we considered all enrolled patients in the current study who had >1 serologic test performed (similar to the methods of the previous study), a similar number of patients (10 [16.9%] of 59) had >1 positive result. Therefore, no clear differences between the 2 studies emerge, despite their differences in methods.

Because coccidioidal serologic tests may be insensitive to early infection (*7*), we attempted to maximize identification of coccidioidomycosis by requiring a second specimen from the convalescent phase of the disease. Paired serologic testing was intended to eliminate the potential for false-negative and false-positive results. Of the 59 patients enrolled in our study, 3 had initial positive results for IgM by EIA but negative results for IgG by EIA and negative results by CF and ID. These 3 patients did not return for a second set of serologic tests; thus, their data (and those of several other patients who did not complete paired serologic testing for unknown reasons) were excluded from further analysis. The 3 patients positive for IgM by EIA but negative for IgG by EIA, CF, and ID may have had coccidioidomycosis. However, Crum et al. (*8*) reported that 18% of positive IgM EIA results without other serologic corroboration may be false-positive results, whereas Blair and Currie found no false-positive results in a similar cohort (*9*). In the present study, the second serologic test resulted in subsequent diagnosis of coccidioidal infection in 6 patients for whom the infection had not been identified by the initial serologic evaluation. Although the requirement for paired serologic testing made it exceedingly challenging to recruit participants and complete the study, the need for definitive serologic diagnosis was paramount.

At the time of the initial evaluation, we sought to identify and delineate signs and symptoms, laboratory findings, and characteristics of patients that would help predict coccidioidal CAP. We confirmed that rash, a symptom known to be associated with coccidioidal infection (*8*), was strongly suggestive of coccidioidal infection in patients with CAP. In contrast, Valdivia et al. (*5*) identified myalgia as the only distinguishing clinical characteristic. Perhaps because of the small number of patients available for analysis in our study, we found that patterns on chest radiographs or other laboratory findings did not enable us to differentiate between patients with and without coccidioidal infection. There was no statistically significant association between coccidioidal infection and type of occupation or type of outdoor recreation activity. Likewise, we could not identify any statistically significant association between coccidioidomycosis and number of years of residence in the disease-endemic area, as had been noted (*10*).

Several challenges limited enrollment of participants in this study. Most common was the issue of the second (or follow-up) serologic test. Many patients were initially willing to participate, but most were unable or unwilling to return for a second serologic test and were thus not enrolled. Even among those who signed a consent form, completed a questionnaire, and provided an initial blood sample, the difficulty of returning for blood collection 6–8 weeks later was shown by the large number of exclusions (24 of 59) because of a lack of paired samples. Soon after the study started, a second challenge arose among the patients whose physicians had already ordered serologic tests. In our institution, EIA results are reported within 1 day of test submission. Thus, a patient’s knowledge of this initial serologic result (whether positive or negative) often led to an unwillingness to participate. Ultimately, we revised the inclusion criteria.

Although a sizeable proportion of patients with CAP had coccidioidomycosis, we almost certainly underestimated its true frequency. We did not systematically attempt to isolate *Coccidioides* spp. from sputum or other respiratory specimens. Moreover, because the sensitivity of available coccidioidal serologic tests is <100%, some patients with coccidioidal CAP may not have been identified. In addition, we did not enroll patients whose clinical evaluation had already included coccidioidal serologic testing. Despite these known preselection biases, we identified coccidioidal infection in 17% of persons with CAP for whom no suspicion of coccidioidomycosis was present initially. We speculate that this finding is an underestimate of the true percentage of coccidioidomycosis in patients with CAP. The limited value of currently available serologic tests makes the diagnosis of coccidioidomycosis difficult, which will no doubt remain so until serologic tests are improved enough to detect early infection.

Our identification of coccidioidal infection in at least 1 of 6 patients who sought treatment for radiologically confirmed CAP in an area where *Coccidioides* spp. are endemic underscores the likelihood that this infection is a common cause of CAP. We believe that coccidioidomycosis should be strongly considered in the differential diagnosis of all patients with CAP who reside in, or who have recently visited, a disease-endemic area.
